# Tree organ growth and carbon allocation dynamics impact the magnitude and δ^13^C signal of stem and soil CO_2_ fluxes

**DOI:** 10.1093/treephys/tpac079

**Published:** 2022-07-15

**Authors:** Yu Tang, Pauliina Schiestl-Aalto, Matthias Saurer, Elina Sahlstedt, Liisa Kulmala, Pasi Kolari, Kira Ryhti, Yann Salmon, Tuula Jyske, Yiyang Ding, Jaana Bäck, Katja T Rinne-Garmston

**Affiliations:** Bioeconomy and Environment Unit, Natural Resources Institute Finland, Latokartanonkaari 9, FI-00790 Helsinki, Finland; Institute for Atmospheric and Earth System Research (INAR)/Forest Sciences, Faculty of Agriculture and Forestry, University of Helsinki, P.O. Box 27, FI-00014 Helsinki, Finland; Institute for Atmospheric and Earth System Research (INAR)/Physics, Faculty of Science, University of Helsinki, P.O. Box 68, FI-00014 Helsinki, Finland; Forest Dynamics, Swiss Federal Institute for Forest, Snow and Landscape Research WSL, Zürcherstrasse 111, 8903 Birmensdorf, Switzerland; Bioeconomy and Environment Unit, Natural Resources Institute Finland, Latokartanonkaari 9, FI-00790 Helsinki, Finland; Institute for Atmospheric and Earth System Research (INAR)/Forest Sciences, Faculty of Agriculture and Forestry, University of Helsinki, P.O. Box 27, FI-00014 Helsinki, Finland; Finnish Meteorological Institute, P.O. Box 503, FI-00101 Helsinki, Finland; Institute for Atmospheric and Earth System Research (INAR)/Physics, Faculty of Science, University of Helsinki, P.O. Box 68, FI-00014 Helsinki, Finland; Institute for Atmospheric and Earth System Research (INAR)/Forest Sciences, Faculty of Agriculture and Forestry, University of Helsinki, P.O. Box 27, FI-00014 Helsinki, Finland; Institute for Atmospheric and Earth System Research (INAR)/Forest Sciences, Faculty of Agriculture and Forestry, University of Helsinki, P.O. Box 27, FI-00014 Helsinki, Finland; Institute for Atmospheric and Earth System Research (INAR)/Physics, Faculty of Science, University of Helsinki, P.O. Box 68, FI-00014 Helsinki, Finland; Production Systems Unit, Natural Resources Institute Finland, Tietotie 2, FI-02150 Espoo, Finland; Department of Forest Sciences, Faculty of Agriculture and Forestry, University of Helsinki, P.O. Box 27, FI-00014 Helsinki, Finland; Institute for Atmospheric and Earth System Research (INAR)/Forest Sciences, Faculty of Agriculture and Forestry, University of Helsinki, P.O. Box 27, FI-00014 Helsinki, Finland; Bioeconomy and Environment Unit, Natural Resources Institute Finland, Latokartanonkaari 9, FI-00790 Helsinki, Finland

**Keywords:** boreal forest, compound-specific, non-structural carbohydrates (NSCs), *Pinus sylvestris*, starch, sucrose, water-soluble carbohydrates (WSCs)

## Abstract

Incomplete knowledge of carbon (C) allocation dynamics in trees hinders accurate modeling and future predictions of tree growth. We studied C allocation dynamics in a mature *Pinus sylvestris* L. dominated forest with a novel analytical approach, allowing the first comparison of: (i) magnitude and δ^13^C of shoot, stem and soil CO_2_ fluxes (*A*_shoot_, *R*_stem_ and *R*_soil_), (ii) concentration and δ^13^C of compound-specific and/or bulk non-structural carbohydrates (NSCs) in phloem and roots and (iii) growth of stem and fine roots. Results showed a significant effect of phloem NSC concentrations on tracheid growth, and both variables significantly impacted *R*_stem_. Also, concentrations of root NSCs, especially starch, had a significant effect on fine root growth, although no effect of root NSC concentrations or root growth was detected on *R*_soil_. Time series analysis between δ^13^C of *A*_shoot_ and δ^13^C of *R*_stem_ or δ^13^C of *R*_soil_ revealed strengthened C allocation to stem or roots under high C demands. Furthermore, we detected a significant correlation between δ^13^C of *R*_stem_ and δ^13^C of phloem sucrose and glucose, but not for starch or water-soluble carbohydrates. Our results indicate the need to include C allocation dynamics into tree growth models. We recommend using compound-specific concentration and δ^13^C analysis to reveal C allocation processes that may not be detected by the conventional approach that utilizes bulk organic matter.

## Introduction

Forests assimilate carbon dioxide (CO_2_) from the atmosphere during photosynthesis, store carbon (C) in tree tissues and release C back to the atmosphere via tree and soil respiration, playing an important role in the global C cycle. However, our knowledge of the C allocation dynamics in trees is incomplete (Hartmann et al. 2020). One of the least understood aspects is not only the interaction between C allocation dynamics and structural growth but also the coupling of the latter with respiration (Darenova et al. 2020). These hinder accurate modeling of tree growth (Guillemot et al. 2017) and thus make it difficult to predict how forest ecosystems will respond to climate change.

A key component of the C balance of trees and forests is stem respiration, which accounts for up to 42% of the aboveground total C budget in mature forests (Waring and Schlesinger 1985). Seasonal variation of stem respiration, which is often derived from stem CO_2_ efflux (*R*_stem_) (Saveyn et al. 2008), is driven mainly by temperature (Amthor 2000) and, albeit less understood, by the intensity of stem growth (Lavigne et al. 2004, Chan et al. 2018). The latter process is highly dependent on C supply from leaves and therefore corresponds to the strength of C allocation via phloem unloading, which is the process of sugar transport from phloem to sink tissues to fuel sink development or resource storage (Milne et al. 2018). There have been studies investigating the linkage between stem respiration and stem growth rate, measured as stem increment rate (Zha et al. 2004, Chan et al. 2018), and the coupling between stem respiration and rate of C allocation to stems (Darenova et al. 2020). However, variation in stem radial increment does not reflect how the C demand changes between tracheid phenophases (Darenova et al. 2020). For example, the rapid tracheid enlargement phase causes most of the diameter increment but requires less C than the secondary wall thickening and lignification stage, which results in less diameter increment (Rossi et al. 2006, Darenova et al. 2020). More accurate quantification of wood formation dynamics can be obtained by analyzing micro-cores (Mäkinen et al. 2008), which, however, is labor intensive and thus rarely applied in C allocation studies (Darenova et al. 2020).

Another key component of the C cycle in forest ecosystems is soil respiration, which is often derived from soil CO_2_ efflux (*R*_soil_). Both components of soil respiration, autotrophic (root and rhizosphere) respiration and heterotrophic respiration, are driven by a seasonally variable supply of recent photosynthates from leaves to belowground (Brüggemann et al. 2011, Hopkins et al. 2013). Belowground C allocation is impacted by, for example, soil temperature (Domisch et al. 2001), soil moisture (Joseph et al. 2020) and soil nutrient availability (Schönbeck et al. 2020). All these factors are linked with root growth (George et al. 1997, Ding et al. 2020). However, the links between root growth, belowground C allocation and soil respiration still remain poorly understood, partly because of the challenges in monitoring root growth dynamics (Perret et al. 2007). The efficiency and ease of determining root growth have been greatly improved by the root scanner method (Dannoura et al. 2008), which nevertheless is still rarely applied (Ding et al. 2020).

A pivotal tool to examine C allocation dynamics in trees is via tracing changes in concentrations of non-structural carbohydrates (NSCs), which serve as both energy carriers and building blocks for amphibolic processes (Hartmann et al. 2020), including growth and respiration. Tracing the seasonal dynamics of NSC concentrations in different organs has improved our understanding of C dynamics at both the whole-tree and ecosystem levels (e.g., Furze et al. 2019, Schiestl-Aalto et al. 2019). Nevertheless, many of the published studies on NSC concentration dynamics have separated NSCs only to its components starch and soluble sugars (e.g., Furze et al. 2019, Davidson et al. 2021), although individual sugars serve distinct functions in trees (Hartmann and Trumbore 2016). For instance, sucrose serves as the predominant transport sugar in trees (Rennie and Turgeon 2009), and thereby changes in sucrose content can provide more accurate information on C allocation compared with changes in total NSC content (Hasibeder et al. 2015).

New insights into C allocation patterns have been obtained from studies tracing stable C isotope composition (δ^13^C) of the assimilates from leaves to respired CO_2_ or tree biomass (Brüggemann et al. 2011). Such studies, sometimes combined with ^13^CO_2_-pulse labeling technique, have identified, for example, the time delay in the coupling of assimilation and *R*_stem_ or *R*_soil_ (Barbour et al. 2005, Wingate et al. 2010) and the dependency of C transfer rate on the phenological stage (Masyagina et al. 2016, Desalme et al. 2017). However, several studies have reported decoupling of δ^13^C signal between respired CO_2_ and assimilates or respiratory substrates (e.g., Brandes et al. 2006, Kodama et al. 2008, Maunoury-Danger et al. 2013). This decoupling is probably because δ^13^C of bulk organic matter, such as total organic matter or water-soluble compounds, was analyzed in these studies, where the distinct functions (Hartmann and Trumbore 2016) and isotopic disparity (Bowling et al. 2008, Rinne et al. 2015) of individual compounds cannot be accounted for. Compared with δ^13^C analysis of bulk matter, compound-specific isotope analysis (CSIA) may provide more accurate knowledge of the temporal dynamics of down-stem allocation of assimilates and its linkage to *R*_stem_ or *R*_soil_. CSIA has been applied together with ^13^CO_2_-pulse labeling to explore C allocation patterns in trees upon changes in environmental conditions, such as elevated CO_2_ and soil warming (Streit et al. 2012) or drought (Galiano et al. 2017). However, despite the advantage of quantifying C fluxes within trees, whole-tree labeling experiments are mostly confined to small-sized trees (Epron et al. 2012), and the environmental and physiological information in the natural abundance variability is lost in the pulse labeling experiments, motivating the current study of using CSIA to trace natural δ^13^C signal in field-grown mature trees.

In this study, we applied a novel methodological approach to explore how organ growth of mature Scots pine (*Pinus sylvestris* L.) interacts with C allocation dynamics during a growing season and how this determines the magnitude and δ^13^C of *R*_stem_ and *R*_soil_ in a boreal forest. For the first time, we combined continuous CO_2_ flux measurements for shoot, stem and soil with high temporal resolution analysis of stem and root growth, which were determined by micro-coring technique and root scanners, respectively. Our interpretations were further supported by data on dimensional growth of shoots and needles and by compound-specific and/or bulk concentration and δ^13^C analysis of NSCs in phloem and roots. We had the following hypotheses:

- HP1: Phloem NSC content is linked to tracheid production rate, and the two factors are coupled with *R*_stem_ dynamics.
- HP2: Root NSC content is linked to root growth rate, and the two factors are coupled with *R*_soil_ dynamics.
- HP3: CSIA provides a better estimate of δ^13^C value of the respiratory substrate, in comparison to bulk isotope analysis, and can identify a coupling between δ^13^C of phloem NSCs and δ^13^C of *R*_stem_.

## Materials and methods

### Site description and environmental data

The study site Hyytiälä SMEAR II station, located in southern Finland (61°51′N, 24°17′E, 170 m a.s.l.), is a managed Scots pine dominated boreal forest stand mixed with Norway spruce (*Picea abies* (L.) Karst) and birches (*Betula pubescens* Ehrh. and *Betula pendula* Roth) in the understory. The Scots pine trees were 56-years-old in 2018. In summer 2016, the stand density for all trees taller than 1.3 m was 1177 ha^−1^, and the dominant height 18 m (Schiestl-Aalto et al. 2019). The soil is a Haplic podzol, with a mineral soil layer depth of 0.5–0.7 m and an average depth of organic layer 5.4 cm (Kulmala et al. 2008, Schiestl-Aalto et al. 2019). The mean annual temperature (T) is 3.5 °C, and the mean annual precipitation 711 mm, almost evenly distributed throughout the year (period 1981–2010, Pirinen et al. 2012).

**Figure 1.**
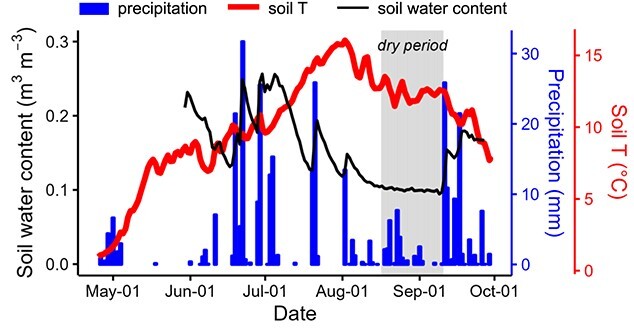
Seasonal courses of soil water content, precipitation and soil temperature (T) in Hyytiälä in the growing season of 2018. The dry period is shaded in gray.

Environmental data in Hyytiälä from 15 April to 30 September 2018 (Figure 1) were obtained from the Smart SMEAR AVAA portal (https://smear.avaa.csc.fi/; see Method S1 available as Supplementary data at *Tree Physiology* Online). During 2018, this site experienced a dry period lasting from 16 August to 11 September (Figure 1), when soil moisture content dropped close to the wilting point and soil water potential below −0.5 MPa in the A horizon, i.e., 2–6 cm depth in the mineral soil.

### Fluxes and δ^13^C of CO_2_ from shoot, stem and soil

Automated chamber systems were implemented for measuring shoot CO_2_ influx (*A*_shoot_), *R*_stem_ and *R*_soil_ from May to October in 2018 (see Table S1 available as Supplementary data at *Tree Physiology* Online). A transparent shoot chamber of 2.1 l was installed in the top canopy of a mature Scots pine tree (‘cuvette tree’), with a debudded 1-year-old shoot inserted (see Figure S1A available as Supplementary data at *Tree Physiology* Online, Kolari et al. 2009). A custom-made transparent stem chamber was built around the stem of the cuvette tree at a height of 15 m (see Figure S1B available as Supplementary data at *Tree Physiology* Online, Rissanen et al. 2020). A transparent soil chamber of 80 l was placed nearby the cuvette tree with all ground vegetation removed (see Figure S1C available as Supplementary data at *Tree Physiology* Online, Aaltonen et al. 2013). The seasonal pattern of *R*_soil_ was verified by manual chamber measurements according to Ryhti et al. (2021), conducted every 2 or 4 weeks from April to September in 2018 on six control plots located nearby, which were with intact tree roots but with all ground vegetation removed.

The shoot chamber was intermittently closed 50–80 times per day for 65 s, the stem chamber 48 times per day for 90 s and the soil chamber eight times per day for 840 s. Sample air was taken to gas analyzers (G2201-I, Picarro, USA) that measured ^12^CO_2_ and ^13^CO_2_ concentrations and replaced by ambient air leaking freely into the chambers. CO_2_ fluxes were calculated via non-linear regression fitted to concentrations of ^12^CO_2_ and ^13^CO_2_ (Kolari et al. 2012) during the first 5–50 s, 10–40 s and 40–200 s after the closing of the shoot, stem and soil chamber, respectively, to exclude transient (pressure pumping) effects in chamber CO_2_. For the soil chamber system, the mass flow effect caused by the differences between the replacement air flow rate and the sample flow rate is insignificant, as discussed in Pumpanen et al. (2001). The C-isotope composition of CO_2_ fluxes was determined from the ratio of ^13^CO_2_ flux to ^12^CO_2_ flux. All isotope data in this paper are reported in δ-notation, in per mil, relative to the international Vienna-Pee Dee Belemnite (V-PDB) standard, defined as

(1) \begin{equation*} {\delta}^{13}C=\left(\frac{R_{\mathrm{sample}}}{R_{\mathrm{standard}}}-1\right)\bullet 1000, \end{equation*}

where ${R}_{\mathrm{sample}}$ and ${R}_{\mathrm{standard}}$ are the ^13^C/^12^C ratios in a sample and standard, respectively. The δ^13^C values of CO_2_ were calibrated by reference CO_2_ gases (Air Liquide, Houston, USA) with δ^13^C of −19 and −3.1‰.

Mean daytime *A*_shoot_, nighttime *R*_stem_ and daily *R*_soil_ were calculated. Nighttime *R*_stem_ was used in subsequent analysis to avoid the influence of the upward transport of stem respired CO_2_ via sap streams at daytime (Teskey et al. 2008) and the depression of daytime *R*_stem_ due to low water status (Saveyn et al. 2007). The impact of nighttime sap flow on *R*_stem_ should be negligible, as the magnitude of nighttime sap flow of the cuvette tree is small (data not shown). As the contribution of CO_2_ released from xylem storage pools to *R*_stem_ was suggested to be insignificant (McGuire and Teskey 2004, Saveyn et al. 2008), its impact on *R*_stem_ and δ^13^C of *R*_stem_ was not considered here. Nighttime was defined as the time when photosynthetically active radiation above the canopy was less than 30 μmol m^−2^ s^−1^ (Kolari et al. 2006). Flux weighted δ^13^C of *A*_shoot_, *R*_stem_ and *R*_soil_ were used in subsequent analysis.

### Concentration and δ^13^C analysis of NSCs

#### Sampling, extraction and purification of WSCs and starch

Stem phloem at 1.3-m height was collected from five mature trees nearby the cuvette tree with a 2-cm diameter corer six times: twice in May and once per month from June to September (see Table S1 available as Supplementary data at *Tree Physiology* Online). Fine roots (<2 mm) were excavated from locations about 100 m away from the cuvette tree but within the same stand. This was done in order to avoid any disturbance to the soil or harm to the roots of our study tree and to prevent any disturbance to other on-going observations conducted at this site. In the root sampling spots, the tree and soil characteristics equal those at our main study site. Roots were sampled from three random spots (see Table S1 available as Supplementary data at *Tree Physiology* Online), approximately 5–15 cm depth from the soil 11 times from May to October every 2 or 4 weeks. All samples were microwaved at 600 W for 1 min within 2 h to stop enzymatic and metabolic activities (Wanek et al. 2001). Samples were then dried and homogenized into a fine powder.

Extraction and purification of water-soluble carbohydrates (WSCs) were performed according to Wanek et al. (2001) and Rinne et al. (2012). In brief, water-soluble compounds were extracted in a water bath at 85 °C for 30 min, and the separated supernatant was purified by three types of sample preparation cartridges (Dionex OnGuard II H, A and P, Thermo Fisher Scientific, Waltham, MA, USA). The purified WSC samples were subsequently freeze-dried, dissolved in 1 mL of Milli-Q water and filtered through a 0.45-μm syringe filter.

Starch was extracted from the pellet of the hot water extraction by enzymatic hydrolysis (Wanek et al. 2001, Lehmann et al. 2019). In brief, lipids were washed out by a sequence of methanol/chloroform/water (12:5:3, v/v/v) solution and Mill-Q water. Starch in pellets was gelatinized at 99 °C for 15 min and hydrolyzed with purified (by Vivaspin 15R, Sartorius, Göttingen, Germany) α-amylase (EC 3.2.1.1, Sigma-Aldrich, Buchs, Switzerland) solution at 85 °C for 2 h in a water bath. The hydrolyzed starch in supernatant was cleaned by centrifugation filters (Vivaspin 500, Sartorius, Göttingen, Germany). WSC and starch samples were stored at −20 °C until isotope analysis.

#### Concentration and δ^13^C analysis of WSCs and starch

Aliquots of WSC and starch samples were pipetted into individual tin capsules (5 × 9 mm, Säntis, Teufen, Switzerland), freeze-dried and wrapped. Concentrations of WSCs were determined using the weights of samples in tin capsules and the weights of plant materials used for extraction. Similarly, starch content was calculated using a conversion factor accounting for the efficiency of the enzymatic conversion of starch to hydrolyzed glucose (see Method S2 available as Supplementary data at *Tree Physiology* Online). δ^13^C values of WSCs and starch were measured using an elemental analyzer (Europa EA-GSL, Sercon Limited, Crewe, UK) coupled to an isotope ratio mass spectrometry (20–22 IRMS, Sercon Limited, Crewe, UK) at the Stable Isotope Laboratory of Luke (Helsinki, Finland). The δ^13^C values of bulk samples were calibrated against IAEA-CH3 (cellulose, −24.724‰), IAEA-CH7 (polyethylene, −32.151‰) and in-house (sucrose, −12.22‰) reference materials. Measurement precision was 0.1‰ (SD), determined from repeat measurement of a quality control material.

#### Concentration and δ^13^C analysis of individual sugars

Compound-specific concentration and isotope analysis of WSCs was done online using a Delta V Advantage IRMS coupled with high-performance liquid chromatography (HPLC) with a Finnigan LC Isolink interface following Rinne et al. (2012) at the Stable Isotope Research Laboratory of WSL (Birmensdorf, Switzerland). Four sugars or sugar alcohols with concentrations above 20 ng C μl^−1^ were detected for HPLC-IRMS δ^13^C analysis: sucrose, glucose, fructose and pinitol/myo-inositol. A dilution series (20, 40, 60, 90, 120 and 180 ng C μl^−1^) of external compound-matched standard solutions (mixture of sucrose, glucose, fructose and pinitol) were analyzed between every 10 samples to calculate sample concentrations and to correct δ^13^C values (Rinne et al. 2012). Only δ^13^C values of sucrose and glucose were used in this research, due to the uncertainties in fructose δ^13^C results and the relatively insignificant temporal variation in pinitol δ^13^C signal (Rinne et al. 2015). The measurement precision is SD <0.35‰ for sucrose standards (−25.370‰) and SD <0.46‰ for glucose standards (−10.240‰).

### Growth data

#### Shoot and needle growth

To trace the timing of shoot and needle growth, length increment of 15 shoots from three trees and the length of one needle of each shoot were measured two or three times a week during 2018, according to Schiestl-Aalto et al. (2013). The observed trees included the cuvette tree and two other trees nearby with similar height and canopy appearance (see Table S1 available as Supplementary data at *Tree Physiology* Online). The monitored shoots were at the top or the middle of the crown. The percentage of shoot and needle length to their final length was calculated for each observation, and the averages were presented.

#### Stem growth

To trace the timing of stem growth, micro-cores (diameter 2 mm, length 15 mm) were collected using Trephor corer (Costruzioni Meccaniche Carabin C., Belluno, Italy). Micro-cores were sampled from five mature trees nearby the cuvette tree (see Table S1 available as Supplementary data at *Tree Physiology* Online), to avoid damage to the cuvette tree. Micro-cores were extracted at 1.3-m height once or twice a week from May to early August, and once every 2 weeks thereafter till October 11. In laboratory, micro-core sections were prepared and analyzed according to Jyske et al. (2014) to determine the number of current-year tracheids in the enlargement phase (a), wall-thickening and lignification phase (b) and mature phase (c). The number of tracheid cells in each phenophase was normalized using the tree-ring width of the previous year (see Method S3 available as Supplementary data at *Tree Physiology* Online, see Figure S2 available as Supplementary data at *Tree Physiology* Online). The Gompertz fitting function (Zeide 1993) was then applied to the number of total tracheids and the number of tracheids at phase c, respectively, using the ‘nlsLM’ function of R package ‘minpack.lm’ (Elzhov et al. 2010). By subtracting the Gompertz fitted number of tracheids at phase c from that of total tracheids, we obtained the daily number of tracheids at phases a and b. The increased number of tracheids at phases a and b relative to the previous day was defined as the growth rate of tracheids.

#### Root growth

Daily growth data of Scots pine roots during 2018 in the study site were published in Ding et al. (2020) and were used here for defining root growth periods. In brief, root elongation was monitored by three flatbed computer scanners (Epson Perfection V37/V39, Seiko Epson, Tokyo, Japan) installed vertically in soil. Scanner 1 was installed in May 2017, and scanner 2 and 3 in April 2018. Daily images captured by the scanners were analyzed by WinRHIZO TRON 2015a software (Regent Instruments Inc., Quebec, Canada) to determine the daily growth of fibrous (absorptive) roots, a type of fine roots chiefly used in the absorption of water and nutrients (Polverigiani et al. 2011). The sum of daily elongation of active fibrous roots per scanner was used in subsequent analysis. More details about scanner installation and image analysis are described in Ding et al. (2020).

### Data analysis

We examined the dependence of: (i) tracheid growth on phloem NSC concentration. Since both variables were performed repeatedly on the same sampling trees, a linear mixed-effects model was applied for analysis (i), using R package ‘NLME’ (Pinheiro et al. 2021), with sampling tree identifier used as a random term. (ii) *R*_stem_ on tracheid growth and phloem NSC concentration. Because *R*_stem_ was measured on a different tree from which tracheid growth and phloem NSC concentration were measured, linear models were used with average values of the independent variables. To remove the effect of temperature on *R*_stem_, we used the residual of *R*_stem_ from the exponential fitting between nighttime *R*_stem_ and temperature (Saveyn et al. 2007) as the response variable. Chan et al. (2018) have found that stem respiration consistently lags behind stem growth by 1 day for mature Scots pine at our study site, as within-stem diffusion resistances cause *R*_stem_ to lag actual stem respiration. Thereby, the response variable, *R*_stem_ residuals, was shifted by 1 day to account for this lagged response. Interaction between tracheid growth and phloem NSC concentration was not significant, thus removed from the final model. (iii) δ^13^C of *R*_stem_ on δ^13^C of phloem NSCs. δ^13^C of *R*_stem_ was tested against the average δ^13^C values of individual phloem NSCs from five trees using linear models. (iv) root growth on root NSC concentration. As no repeated measurements were done for root NSC concentration, root growth and *R*_soil_, we applied linear models with average values of the response variables and the independent variables, assuming that the average values represent the conditions of the stand. To remove the temperature and soil moisture effects on growth of fibrous root growth, we tested the effect of root NSC concentration on the residual of the regression between root growth and soil temperature and soil moisture (Ding et al. 2020). (v) *R*_soil_ on root growth and root NSC concentration. As in (iv), we tested the residual of the regression between *R*_soil_ and soil temperature and soil moisture against the independent variables: the residuals of fibrous root growth (see iv) and concentrations of root NSCs. For all analyses from (i) to (v), time (day of year, DOY) was originally included as a covariate and removed from the final model when not having a significant effect on the response variable.

To test the temporal variability in the correlation between δ^13^C of *A*_shoot_ and δ^13^C of *R*_stem_ or δ^13^C of *R*_soil_, we applied the wavelet coherence analysis, using the R package ‘WaveletComp’ (Rösch and Schmidbauer 2016). Wavelet coherence analysis can not only determine the multi-temporal correlations between two time series but also quantify the phase differences (or time lags) between the two time series (Vargas et al. 2011). This method has been applied in *R*_soil_ (Vargas et al. 2011) or *R*_stem_ (Chan et al. 2018) studies, because of its advantage in analyzing nonstationary and heteroscedastic time series signals. Thereby, this approach is robust for time series signals with unexpected noises, rising from, e.g., rain pulses or heat waves. Here, we determined correlation and phase difference (or time lag) between daily δ^13^C of *A*_shoot_ and daily δ^13^C of *R*_stem_ or δ^13^C of *R*_soil_ at different time periods or frequencies. Any gaps (2%) in the input data were linearly interpolated. The output results include cross-correlation between *x* (daily δ^13^C of *A*_shoot_) and *y* (daily δ^13^C of *R*_stem_ or δ^13^C of *R*_soil_) time series at any given time period (or frequency) and phase difference (or time lag) of *x* over *y*. As δ^13^C signal is transported from photosynthesis to respiration, we considered only the phase differences when δ^13^C of *A*_shoot_ led δ^13^C of *R*_stem_ or δ^13^C of *R*_soil_ as meaningful.

All statistical analysis was done in R version 4.0.0 (R Core Team 2020).

**Figure 2.**
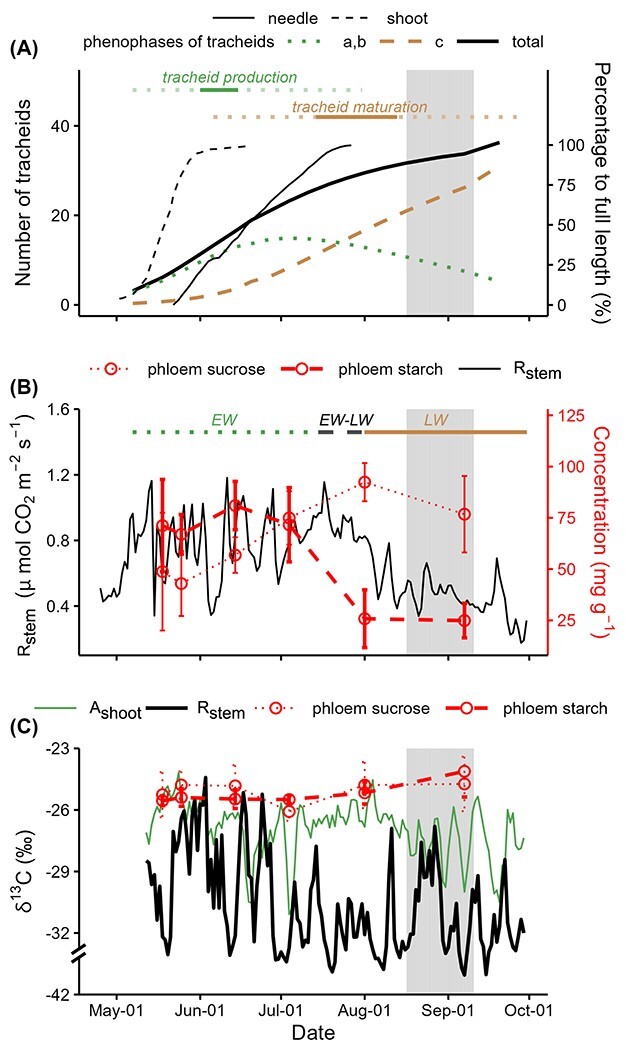
Seasonal course of organ growth, magnitude and δ^13^C of stem CO_2_ efflux (*R*_stem_) and concentration and δ^13^C of phloem NSCs of *Pinus sylvestris* L. during the 2018 growing season. (A) The number of tracheids at different phenophases together with needle and shoot length development relative to their full length. For the phenophases of tracheids, `a' indicates the radial cell enlargement, `b' the secondary cell wall deposition and lignification and `c' the cell maturation. The active tracheid production period is indicated by a dashed green line, the maximum tracheid production period by a solid green line, the tracheid maturation period by a dashed brown line, and the maximum tracheid maturation period by a solid brown line. (B) Daily Rstem together with sucrose and starch concentration in phloem. Earlywood (EW) growth period is indicated by a dotted green line; latewood (LW) growth period by a solid brown line; EW-LW transition period by a dashed black line. (C) δ^13^C of *R*_stem_, shoot CO_2_ influx (A_shoot_), phloem sucrose and phloem starch. Error bars represent the standard deviation. The dry period is shaded in gray.

## Results

### HP1—Relationships between tracheid growth, phloem NSC content and R_stem_

Tracheid growth periods were defined as follows (Figure 2A): tracheid production period (May 7 to July 31), tracheid maturation period (June 6 to October 11), period with maximum tracheid production rate (June 1 to June 15) and period with maximum tracheid maturation rate (July 14 to August 13), according to the Gompertz fitted tracheid production and maturation curves (see Method S3 available as Supplementary data at *Tree Physiology* Online). Earlywood (EW) growth period of year 2018 was from May 7 to July 14, latewood (LW) growth period from August 2 to October 11 and EW-LW transition period from July 15 to August 1 (Figure 2B, see Method S4 available as Supplementary data at *Tree Physiology* Online).

During the EW-LW transition period, between July 4 and August 1, a dramatic depletion of phloem starch content from 72 to 26 mg g^−1^ and an increase in phloem sucrose content were observed, indicating significant re-mobilization of phloem starch during this period (Figure 2B). As starch accumulated over a period and had rather stable δ^13^C values which differed from that of glucose and sucrose (Figure 2B and C), the degradation of starch would very likely blur the environmental signal imprinted in δ^13^C of phloem sugars. Thereby, August 1 was excluded from the linear models for testing the correlation between δ^13^C of *R*_stem_ and δ^13^C of individual phloem NSCs (Table 1).

**Table 1:** Results of linear models for testing the dependence of δ^13^C of stem CO_2_ efflux (*R*_stem_) on δ^13^C of different phloem NSC pools, i.e., sucrose, glucose, starch and WSCs. Average δ^13^C values of phloem NSCs from five trees were used in the models. Data from four observation days were used in the models.

Response variable (*Y*)	δ^13^C of *R*_stem_			
Independent variable (*X*)	δ^13^C of sucrose	δ^13^C of glucose	δ^13^C of starch	δ^13^C of WSCs
*P*-value for *X* (Symbol of estimate)	0.02 (+)	0.01 (+)	0.41 (+)	0.22 (+)
Model *R*^2^	0.96	0.98	0.35	0.61

The nighttime *R*_stem_ increased when tracheid production initiated and decreased during the EW-LW transition period and LW maturation period (Figure 2A and B), showing a synchrony with air T. *R*_stem_ varied between 0.1 and 1.2 μmol CO_2_ m^−2^ s^−1^ during the entire period of study, in agreement with the earlier reports (<1 μmol m^−2^ s^−1^ in general) by Kolari et al. (2009) for the study site.

Tracheid growth rate was positively correlated with the concentration of glucose, negatively to that of sucrose in phloem (Table 2). Tracheid growth rate had a positive linear regression with *R*_stem_ residuals (Table 3), whereas phloem NSC concentration showed diverse effects on *R*_stem_ residuals (Table 3). For instance, concentration of phloem sucrose had a negative correlation with *R*_stem_ residuals (*P* = 0.02). In contrast, phloem starch concentration was positively correlated with *R*_stem_ residuals (*P* = 0.01).

**Table 2:** Results of linear mixed-effects models for testing the dependence of tracheid growth rate (*G*_tracheid_) on concentration of different phloem NSC pools, i.e., sucrose (C_Suc_), glucose (C_Glu_), starch (C_Sta_), water-soluble carbohydrates (C_WSC_) and total NSCs (C_NSC_). Random effect of the mixed-effects models was sampling tree identifier. DOY was included in the models as a covariant. Sample size were 30, and degrees of freedom 23.

Response variable (*Y*)	*G*_tracheid_				
Independent variable (*X*)	C_Suc_	C_Glu_	C_Sta_	C_WSC_	C_NSC_
*P*-value for *X* (Symbol of estimate)	0.04 (−)	0.01 (+)	0.65 (−)	0.08 (−)	0.12 (−)
*P*-value for DOY (Symbol of estimate)	<0.001 (−)	<0.001 (−)	<0.001 (−)	<0.001 (−)	<0.001 (−)
Model *R*^2^	0.80	0.79	0.77	0.79	0.79

**Table 3:** Results of linear models for testing the dependence of stem CO_2_ efflux (*R*_stem_) on tracheid growth rate (*G*_tracheid_) and concentration of different phloem NSC pools, i.e., sucrose (C_Suc_), glucose (C_Glu_), starch (C_Sta_), water-soluble carbohydrates (C_WSC_) and total NSCs (C_NSC_). Average concentrations of phloem NSC from five trees were used in the models. Data from six observation days were used in the models.

Response variable (*Y*)	*R*_stem_ residuals					
Independent variable (*X*)	*G*_tracheid_	C_Suc_	C_Glu_	C_Sta_	C_WSC_	C_NSC_
*P*-value for *X* (Symbol of estimate)	0.02 (+)	0.02 (−)	0.08 (+)	0.01 (+)	0.10 (−)	0.67 (+)
Model *R*^2^	0.79	0.77	0.57	0.84	0.53	0.05

**Figure 3.**
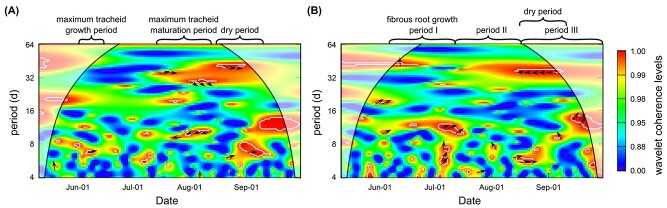
Wavelet coherence analysis and phase difference between (A) δ^13^C of shoot CO_2_ influx (A_shoot_) and δ^13^C of stem CO_2_ efflux (R_stem_), (B) δ^13^C of A_shoot_ and δ^13^C of soil CO_2_ efflux (R_soil_) in Hyytiälä during the growing season of 2018 at daily intervals for any given time periods (*y* axis). Coherence levels are indicated by the colors from blue (low values) to red (high values). White contours represent the 5% significance level, and shaded white regions indicate the region that is potentially impacted by edge effects. Phase differences (i.e., time lags) when δ^13^C of A_shoot_ leads δ^13^C of R_stem_ or R_soil_ with significant wavelet coherence are indicated by arrows. Arrows pointing right indicate no lag; down, ~0.25 period lag; and left, ~0.5 period lag.

The temporal correlation between δ^13^C of *A*_shoot_ and δ^13^C of *R*_stem_ via wavelet coherence analysis is shown in Figure 3A. At any time period, the regions with significant wavelet coherence (red areas within the white contours) were not continuous throughout the observation period, with gaps existing in between. It denotes that temporal correlations between δ^13^C of *A*_shoot_ and δ^13^C of *R*_stem_ were not constant throughout the growing season. The phase differences when δ^13^C of *A*_shoot_ led changes in δ^13^C of *R*_stem_ with significant coherence levels (marked by arrows) mainly appeared in the maximum tracheid growth period, maximum tracheid maturation period and dry period. It indicates intensified correlations between δ^13^C of *A*_shoot_ and δ^13^C of *R*_stem_ during these periods, compared with other periods.

### HP2—Relationships between fibrous root growth, root NSC content and R_soil_

**Figure 4.**
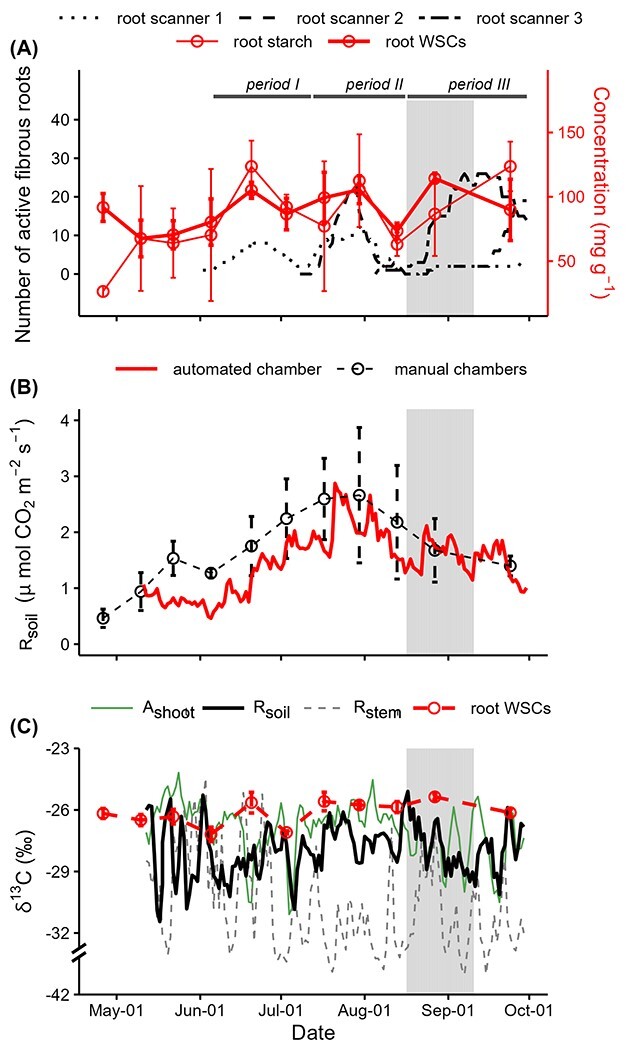
Seasonal course of fibrous root growth, magnitude and δ^13^C of soil CO_2_ efflux (*R*_soil_) and concentration and δ^13^C of root NSCs of *Pinus sylvestris* L. during the 2018 growing season. (A) The number of active fibrous roots measured by the three root growth scanners together with the concentrations of root WSCs and root starch. Fibrous growth period I (June 6 to July 1 2), II (July 13 to August 16) and III (August 17 to September 30) are indicated by black lines. (B) R_soil_ measured by the automated chamber and by the manual chambers. (C) δ^13^C of R_soil_, shoot CO_2_ influx (A_shoot_), stem CO_2_ efflux (R_stem_) and root WSCs. Error bars represent the standard deviation. The dry period is shaded in gray.

Three fibrous root growth periods were identified (Figure 4A): period I (June 6 to July 12), period II (July 13 to August 16) and period III (August 17 to September 30), according to changes in the number of active fibrous roots captured by root growth scanners. The fibrous root flush in period I was captured by scanner 1 but not by scanner 2 or 3. This scanner-specific fibrous root growth pattern in period I may be associated with the fact that scanners 2 and 3 were not installed until April of the study year, whereas the installation of scanner 1 in May 2017 enabled undisturbed root growth in the observation spot. Both scanners 1 and 2 captured the fibrous growth in period II, whereas root growth around scanner 3 might still have been disturbed by the installation of scanner. The fibrous growth in period III was significantly recorded only by scanner 3, reflecting a spatial heterogenous growth pattern initiated by water stress (Ding et al. 2020). For all three root growth periods, the root growth patterns were to some extent impacted by soil heterogeneity.

Root NSC concentration shared a similar seasonal pattern with fibrous root growth (Figure 4A). In period I of fibrous root growth, which initiated directly after shoots had developed to their full length (Figure 2A), the maximum number of active fibrous roots corresponded to the first peak in root NSC concentration. Simultaneous increase in fibrous root growth and root NSC content was observed also for period II and for period III, which included the dry period (Figure 4A). During the entire studied period, root starch content varied in a similar manner to root WSC content (Pearson *r =* 0.64, *P =* 0.05, Figure 4A).

Both the absolute values and seasonal variation of *R*_soil_ captured by the automated chamber were in agreement with that observed by the manual chambers (Figure 4B). The daily *R*_soil_ captured by the automated soil chamber varied from 0.5 to 2.9 μmol CO_2_ m^−2^ s^−1^ during the observation period (Figure 4B). *R*_soil_ peaked at the end of July, coinciding with the peaks of fibrous root growth and root NSC content in period II.

Fibrous root growth and root NSC concentrations had no significant effect on *R*_soil_ residuals. However, positive linear relationships existed between root NSC concentrations and residuals of fibrous root growth (Table 4). The linear model with root starch concentration explained more of the variance than the other models in Table 4.

**Table 4:** Results of linear models for testing the dependence of fibrous root growth rate (*G*_fibrous_) residuals on concentration of different root NSC pools, i.e., starch (C_Sta_), water-soluble carbohydrates (C_WSC_) and total NSCs (C_NSC_). Average NSC concentration of roots collected from three random spots, and average G_fibrous_ residuals measured by three root scanners were used in the models. Data from nine observation days were used in the models

Response variable (*Y*)	G_fibrous_ residuals		
Independent variable (*X*)	C_Sta_	C_WSC_	C_NSC_
*P*-value for *X* (Symbol of estimate)	0.004 (+)	0.20 (+)	0.006 (+)
Model *R*^2^	0.71	0.22	0.69

The temporal dynamics of C allocation to soil was tested by wavelet coherence analysis between δ^13^C of *A*_shoot_ and δ^13^C of *R*_soil_ (Figure 3B). The temporal correlations between the two variables were not constant over the growing season at any time period, as indicated by the gaps between the regions with significant coherence in Figure 3B. The phase differences when δ^13^C of *A*_shoot_ led δ^13^C of *R*_stem_ (marked by arrows) mainly appeared in the fibrous root growth period I and period III, which included the dry period. It indicates a tighter relationship between δ^13^C of *A*_shoot_ and δ^13^C of *R*_stem_ for these periods than other periods.

### HP3 and seasonal variations in δ^13^C of R_stem_ and R_soil_

Over the observation period, daily δ^13^C of *R*_stem_ varied 18.7‰, whereas δ^13^C of phloem WSCs and δ^13^C of phloem sucrose varied only 0.9 and 1.3‰, respectively (Figure 2C). δ^13^C of *R*_stem_ exhibited a gradual decline from early June to early August, with extremely low values from mid-July onward, coinciding with the EW-LW transition period of stem growth (Figure 2B and C). *R*_stem_ was overall more ^13^C-depleted relative to *A*_shoot_, phloem sucrose and phloem starch by 4.5, 6.4 and 6.2‰, respectively (Figure 2C). δ^13^C of *R*_stem_ was significantly correlated with δ^13^C of sucrose (*P* = 0.02) and glucose (*P* = 0.01), but not with δ^13^C of WSCs or starch in phloem (Table 1).

Similar to δ^13^C of *R*_stem_, the range of δ^13^C variation of *R*_soil_ (11.7‰) was much higher than that of root WSCs (1.8‰) during the studied period (Figure 4C). δ^13^C of *R*_soil_ exhibited a progressive decrease of 5.6‰ during the dry period, coinciding with a decreasing trend in δ^13^C of *A*_shoot_ which started before the dry period (Figure 4C). Overall, δ^13^C of *R*_soil_ was on average 1.0 and 1.8‰ lower than δ^13^C of *A*_shoot_ and δ^13^C of root WSCs, respectively. δ^13^C of *R*_soil_ was not significantly correlated with δ^13^C of root WSCs, even though a time lag from 0 to 20 days was considered (not shown).

## Discussion

To the best of our knowledge, we present the first quantification of the interaction between NSC pools, organ growth and respired CO_2_ of stem and roots based on field observation of mature field-grown trees. We identified (i) negative correlations between phloem sucrose concentration and tracheid growth and *R*_stem_ residuals, (ii) a positive correlation between concentration of root starch and fibrous root growth rate, (iii) strengthened couplings between δ^13^C of *A*_shoot_ and δ^13^C of *R*_stem_ or *R*_soil_ under high stem or root growth demand periods and (iv) a significant correlation between δ^13^C of *R*_stem_ and δ^13^C of phloem sucrose and glucose, but not for starch or WSCs. Our results provide evidence that C allocation dynamics in trees are highly coupled with growth demands of sink organs via regulations on NSC pools, and suggest that a better knowledge about these dynamics would improve the current presentation of C allocation scheme in land surface models.

### HP1—Phloem sucrose dynamics as an indicator of phloem unloading strength

Concentrations of different phloem NSC pools have diverse relationships with tracheid growth and *R*_stem_ (Tables 2 and 3), among which the most intriguing finding is the negative correlations between sucrose concentration and tracheid growth and *R*_stem_ residuals. Sucrose, as the predominant transport sugar in trees (Rennie and Turgeon 2009), is often converted to hexoses in sink cells via sucrose synthase or invertase to increase phloem unloading rate (Roitsch et al. 2003, Stein and Granot 2019). Conversely, as a result of reduced sucrose synthase or invertase activity, high sucrose concentration in sink cells may indicate a curtailed phloem unloading rate. The significant role of sucrose cleavage in long-distance assimilate transport, associated with both enzyme mechanisms, has been shown in controlled experiments on herbaceous plants or saplings using transgenic approach (Roitsch et al. 2003, Dominguez  et al. 2021). The observed negative correlations between phloem sucrose concentration and tracheid growth in our study thereby support that phloem unloading rate increases under high tracheid growth rate, providing probably the first piece of evidence from field-grown mature trees. The decreased phloem sucrose content, which can be interpreted as increased phloem unloading rate (or C allocation rate) to stem, is apparently due to high C demands of tracheids at enlargement, thickening and lignification phases for completing maturation (Darenova et al. 2020). This result is also in line with the previous finding that C availability influenced wood formation based on phloem girdling experiments (Winkler and Oberhuber 2017).

Wavelet coherence analysis between δ^13^C of *A*_shoot_ and δ^13^C of *R*_stem_ (Figure 3A) further supports that C allocation to stem is intensified under high demands. First, the inconsistency of wavelet coherence between the two signals demonstrates the dynamic nature of C allocation over the growing season. Indeed, the down-stem transport of carbohydrates operates at very different velocities depending on environmental conditions and sink demand (Kuzyakov and Gavrichkova 2010). Second, the phase differences with significant coherence levels appeared predominantly in the maximum tracheid growth period, the maximum tracheid mature period and the dry period. All these periods were under high C demands either for tracheid growth (Darenova et al. 2020) or for coping with water stress (Chaves et al. 2002). This result suggests that C allocation dynamics as a function of sink demands, for example, via a dynamic growth model (Schiestl-Aalto et al. 2015), can be used in land surface models. Currently, the C allocation schemes in the land surface models are set as functions of net primary production, which generates large uncertainties in long-term estimates of aboveground biomass accumulation (Montané et al. 2017).

Our results further denote that the increase in C allocation rate to stem and associated enhanced tracheid growth rate promote *R*_stem_, since both phloem NSC concentrations and tracheid growth have significant effects on *R*_stem_ (Table 3). Likewise, Maier et al. (2010) observed a linear correlation between stem carbohydrate content and temperature corrected *R*_stem_ for girdled loblolly pine trees. Chan et al. (2018) revealed a significant correlation between tracheid growth rate and stem growth respiration, based on modeled growth signal from dendrometer measurements. However, we note that the correlations are negative, albeit not significant, if concentrations of phloem WSCs are used in the models (Tables 2 and 3). On one hand, this negative correlation may rise from the above-mentioned adverse effect of sucrose, which constitutes a significant portion (34%) of WSCs in phloem. On the other hand, it points out potential confusions that concentration analysis on WSCs may cause in in-depth understanding of C allocation dynamics, tracheid growth and stem respiration. Taken together, our results demonstrate that compound-specific concentration analysis of NSCs can provide new insights on the control of sink metabolic activity on tree C allocation (Gessler 2021).

### HP2—Root starch as a dynamic C pool supporting root growth

Starch in sink tissues has been long regarded as the primary ‘old’ reserve, the counterpart of newly assimilated C, used to buffer growth demands and photosynthetic supply (Kozlowski 1992, Vincent-Barbaroux et al. 2019). However, our observations denote that the root starch mainly originated from the newly assimilated C, as starch content was very low before May and showed a synchrony with WSC content (Figure 4A). It is not likely that starch and WSCs in roots originated from root lipids, considering a similar seasonal pattern in lipids, starch and soluble sugars in roots (Sanz-Perez et al. 2009). Our results further address that root starch serves as a rapid and easily accessible intra-seasonal reserve fueling growth, according to the following facts. First, root starch content presented a larger amplitude of variation than root WSC content (Figure 4A), demonstrating its relatively fast turnover rate. Second, root starch concentration had a significant positive correlation with fibrous root growth residuals (*P =* 0.004, Table 4), in line with the observation for mature Norway spruce (Wang et al. 2018). This finding also aligns with the previous report that fresh assimilates are intricately involved in new root growth of conifers (Villar-Salvador et al. 2015 and reference therein). Overall, the results clearly support the hypothesized coupling between root growth and C allocation to roots.

However, we did not find a significant effect of root growth or root NSC content on *R*_soil_, though previous studies have suggested that substrate availability in roots can impact *R*_soil_ (Högberg et al. 2001, Hartley et al. 2007) by controlling autotrophic (root and rhizosphere) respiration (Hopkins et al. 2013) and stimulating decomposition of old organic C pool in soil via root exudation (Adamczyk et al. 2019). The observed decoupling is probably due to heterotrophic respiration, which may not respond to increases in root NSC content in a straightforward manner. Notable part of heterotrophic organisms is independent of tree roots, and an increase in root NSC content does not directly mean increased allocation to belowground symbionts nor root exudates. In addition, this decoupling may partly rise from the fact that our observed *R*_soil_ at soil surface does not equal to the CO_2_ production rate in soil, as the former depends also on soil CO_2_ diffusion rate (Fang and Moncrieff 1999).

Nevertheless, the wavelet coherence analysis between δ^13^C of *A*_shoot_ and δ^13^C of *R*_soil_ indicates an intensified C allocation to belowground when C demands of roots increase, notably for the fibrous root growth period I and the dry period. The apparent allocation of new assimilates for the initial fibrous root flush is important to increase the chances of getting access to water before the onset of any potential summer drought (Poot and Lambers 2003). The increased C allocation during the dry period is likely to support water and nutrient acquisition via stronger WSC solution in roots (Figure 4A) or via development of new roots (Zadworny and Eissenstat 2011, Joseph et al. 2020).

### HP3—The choice of the substrate for δ^13^C analysis is crucial for understanding δ^13^C of R_stem_ and R_soil_

The observed ^13^C-depletion of *R*_soil_ compared with root WSCs (Figure 4C) can be partly ascribed to the ^13^C-depletion of root respired CO_2_ (Werth and Kuzyakov 2010 and references therein), considering that root respiration accounts for about half of soil respiration at our site (Ryhti et al. 2021). It can also be attributed to heterotrophic respiration, which has been suggested to be even more ^13^C-depleted than autotrophic respiration for a Scots pine forest in northern Sweden (Bhupinderpal-Singh et al. 2003). Previous field investigations have reported higher or similar δ^13^C values of *R*_soil_ relative to soil organic matter or soil litter (Bowling et al. 2008 and references therein; Wingate et al. 2010). Together with our finding, δ^13^C values of *R*_soil_ reflected the contributions of both newly assimilated C and old soil C stores to soil respiration (Søe et al. 2004). The result further demonstrates that a profound understanding of ^13^C-discrimination during soil respiration processes in future studies will depend on thorough definition of δ^13^C values of substrates for soil respiration, which should include not only soil C but also root NSCs.

Previous studies on the δ^13^C difference between *R*_stem_ and respiratory substrates are contradictory (Damesin and Lelarge 2003, Kodama et al. 2008, Wingate et al. 2010). As was the case for δ^13^C of *R*_soil_, also these studies were based on analysis of a mixture of organic compounds to estimate δ^13^C of respiratory substrates. Wingate et al. (2010) reported a ^13^C-depletion of *R*_stem_ relative to phloem water-soluble organic matter for maritime pine, Damesin and Lelarge (2003) observed a ^13^C-enrichment of *R*_stem_ in comparison to stem total organic matter for European beech and Kodama et al. (2008) obtained higher or comparable δ^13^C of *R*_stem_ relative to phloem exudate organic matter for Scots pine. These contrasting results probably stem from varying δ^13^C signals of different substrates (Bowling et al. 2008). Water-soluble organic matter includes also amino acids, organic acids and phenolic compounds aside from WSCs (Antonova and Stasova 2006), and total organic matter consists of an even bigger mixture of compounds. In contrast, the WSCs analyzed in the present study contain only sugars and sugar alcohols (Rinne et al. 2012), and the sucrose, obtained via CSIA, is the sugar used for down-stem transport (Rennie and Turgeon 2009). Although other storage compounds, such as lipids, can also provide respiratory substrate, these compounds are used when carbohydrate supply from photosynthesis is significantly reduced, e.g., during drought or shading (Fischer et al. 2015, Hanf et al. 2015). Hence our study provides a more accurate estimate for the δ^13^C value of the respiratory substrate under normal conditions. The ^13^C-depletion of *R*_stem_ relative to phloem WSCs and sucrose observed in our data (Figure 2C) may be related to intensive activities of oxidative pentose phosphate (Bathellier et al. 2008) and phospho*enol*pyruvate carboxylase (Gessler et al. 2009). The former decarboxylates the ^13^C-depleted C-1 position of glucose (Rossmann et al. 1991), and the latter refixes CO_2_ against ^12^C (Farquhar 1983), thus enriching the product and depleting the remaining CO_2_.

Also the decoupling between δ^13^C of stem or phloem substrates and δ^13^C of *R*_stem_, which has been reported in previous studies (e.g., Brandes et al. 2006, Kodama et al. 2008, Maunoury-Danger et al. 2013), may be explained by the fact that these studies are based on δ^13^C analysis of a mixture of various types of organic compounds. Using phloem sucrose and glucose as substrates, we observed a tight coupling between δ^13^C of *R*_stem_ and δ^13^C of substrates (Table 1). This coupling further supports HP1, suggesting that phloem transport sugar (i.e., sucrose) provides substrate for stem respiration, in line with findings from ^13^CO_2_-pulse-labeling studies (Epron et al. 2012 and references therein). When using phloem WSCs or starch as substrates, we did not detect such coupling with δ^13^C of *R*_stem_ (Table 1). This is most likely because the δ^13^C signal of WSCs is dampened by the mixing of different components and that of starch by the mixing of assimilated C formed at different times. It also weakens the assumption that phloem water extracts are close to pure sucrose, which grounded the common use of these extracts as a substitute for phloem sucrose in δ^13^C analysis. Therefore, we recommend going beyond the conventional isotope analysis of bulk matter to CSIA, which can provide us more detailed information about the use of individual NSCs for growth and respiration. For example, the use of CSIA can shed light on changes of respiratory substrates, e.g., under stressed conditions or over a diurnal course, and the associated C isotope fractionation during respiration.

### Impact of extremely warm and dry weather on CO_2_ fluxes and growth

Extreme weather events, for example, heatwaves and droughts, can severely affect tree growth (Rosner et al. 2018, Shi et al. 2021) and CO_2_ exchange between trees and the atmosphere (Rodríguez-Calcerrada et al. 2014, Blessing et al. 2016). In the studied year 2018, southern Scandinavia experienced an exceptionally hot and dry summer (Lindroth et al. 2020). Unexpectedly, we recorded limited responses in *R*_stem_ and *R*_soil_ to the extreme weather, which, however, is in line with Lindroth et al. (2020) who studied eddy covariance fluxes at our study site. Also, we did not observe a clear impact of the dry period on the tracheid growth of Scots pine, in accordance with the report by Salomón et al. (2022) that the 2018 drought did not lead to consistent growth reduction even in central Europe. The low impact of the hot and dry weather in year 2018 on tree growth and C exchange fluxes can be attributed to the following reasons. First, the impact of the dry weather, which tends to decrease *R*_stem_ and *R*_soil_ (Rodríguez-Calcerrada et al. 2014, Blessing et al. 2016), may counterbalance the impact of increased temperatures, which promote respiration rate. Second, Scots pine can tolerate moderate drought well (Cregg and Zhang 2001, Baumgarten et al. 2019), and Matkala et al. (2021) reported that the soil moisture stress in 2018 was not severe enough for this species to suffer from it at a site in northern Finland (Matkala et al. 2021). Third, the dry period appeared at the late part of the growing season, when tracheid production had completed (Figure 2A), thus posing no impact on tracheid growth. Taken together, our data imply that the timing, severity and concurrent occurrence of extreme weather events should be carefully evaluated when forecasting tree growth and forest C exchange, especially in regions with a potential shift toward warmer and drier summers, such as Scandinavia (Christidis and Stott 2021).

### Methodological concerns

Only one stem chamber was used to capture *R*_stem_ and δ^13^C of *R*_stem_, which would be insufficient for analyzing absolute values of those parameters. However, when studying processes and their responses to driving factors, such as seasonal variability in *R*_stem_, high time resolution data can provide useful information even with a low number of trees. This is also supported by Tarvainen et al. (2018), who demonstrated the similarity in temporal variations of *R*_stem_ measured from different Scots pine trees growing at the same stand. Furthermore, performance assessment of the chamber system used in our study indicates that it is able to capture small fluxes, such as volatile organic compounds (Rissanen et al. 2020). Moreover, the quality of δ^13^C of *R*_stem_ data was evaluated by changing the calculating methods (see Figure S3 available as Supplementary data at *Tree Physiology* Online). The agreement of the results across calculating methods supported the reliability of our data, although the day-to-day variation in δ^13^C of *R*_stem_ was rather high. We also had only one automated soil chamber to determine *R*_soil_. However, we used manual soil chamber data to validate the seasonal course of *R*_soil_ and found good agreement between the two (Figure 4B).

Root samples were taken from spots away from the cuvette tree, which may lead to deviation in absolute values of concentration and δ^13^C of root NSCs between the measured roots and the roots of the cuvette tree. Nevertheless, also in this case, our statistical analysis relies more on the seasonal variations in concentration and δ^13^C of root NSCs rather than the absolute values. Leavitt and Long (1984) suggested that sampling from four trees would provide accurate site-representative δ^13^C trend and absolute values for tree rings. We assume that pooled root samples from three random spots represent the trends in concentration and δ^13^C of root NSCs for our stand.

The stem chamber for δ^13^C of *R*_stem_ measurement was installed at a higher position than the height of phloem sampling. Previous observation on mature pine trees (Brandes et al. 2006) has shown that δ^13^C of *R*_stem_ does not change with height. Moreover, it would have been optimal to measure NSC contents both for cambium and phloem, when connecting phloem substrates to *R*_stem_ and cambium growth. However, since the cambium sampling is detrimental to trees, it is impractical in the study site intended for long-term observation.

## Conclusions

In this study, we aimed to examine the interaction between tree organ growth and C allocation dynamics on field-grown mature trees. With this aim, for the first time, we traced the magnitude and δ^13^C signals of shoot, stem and soil CO_2_ fluxes with growth measurements of tree organs (i.e., shoots, needles, stem and fine roots), together with compound-specific and/or bulk concentration and δ^13^C analysis of NSCs in phloem and roots of Scots pine (*Pinus sylvestris* L.) trees over a growing season. The results demonstrate that the allocation of newly assimilated C to stem and roots increases under high growth rate of stem and roots, respectively. The pieces of evidence include: (i) phloem sucrose content, which was conversely linked to phloem unloading rate, had a significant negative correlation with tracheid growth; (ii) δ^13^C of phloem sucrose had a significant correlation with δ^13^C of *R*_stem_; (iii) concentration of root starch, which mainly originated from new assimilates, had a significant positive effect on fine root growth and (iv) time-series analysis revealed significant temporal correlations between δ^13^C of *A*_shoot_ and δ^13^C of *R*_stem_ or δ^13^C of *R*_soil_ for the periods when stem or roots were under high C demands. Increased C allocation and associated growth contributed to higher *R*_stem_, which however was not observed for *R*_soil_ likely due to the disturbance of heterotrophic soil respiration. We suggest that the dynamics of C allocation in response to tree organ growth should be considered in models based on whole tree C allocation for projecting forest growth under climate change. Our results also emphasize the need to carefully evaluate the composition of the substrates, which are used for concentration and δ^13^C analysis, to study tree growth and C allocation dynamics. Where possible, compound-specific concentration and δ^13^C analysis should be used, as these signals can provide more detailed and accurate information on C allocation and utilization processes in trees.

## Supplementary Material

SI_tpac079

## Data and materials availability

The data that support the findings of this study are available from the corresponding author upon reasonable request.
