# Supplementary material for: Tree organ growth and carbon allocation dynamics impact the magnitude and δ13C signal of stem and soil CO2 fluxes
Source: Tree Physiol. 2022 Jul 15;42(12):2404–18. doi: 10.1093/treephys/tpac079 (PMC10101690; doi:10.1093/treephys/tpac079)
Supplement: SI_tpac079 [file si_tpac079.docx]

## Supplementary Information

Article title: Tree organ growth and carbon allocation dynamics impact the magnitude and δ^13^C signal of stem and soil CO_2_ fluxes

Authors: Yu Tang, Pauliina Schiestl-Aalto, Matthias Saurer, Elina Sahlstedt, Liisa Kulmala, Pasi Kolari, Kira Ryhti, Tuula Jyske, Yiyang Ding, Yann Salmon, Jaana Bäck, Katja T. Rinne-Garmston

The following Supplementary Information is available for this article:

**Table S1** Information on which trees were used for CO_2_ flux measurements, growth measurements and non-structural carbohydrate (NSC) concentration and δ^13^C analysis.

**Figure S1** The gas exchange measurement chambers used in this study.

**Figure S2** Seasonal course of total number of current-year tracheids of *Pinus sylvestris* L. during the growing season of 2018 in Hyytiälä.

**Method S1** Measurement of environmental data

**Method S2** Calculation of starch concentration from enzymatic hydrolysis

**Method S3** Definition of tracheid production and maturation periods

**Method S4** Definition of earlywood and latewood growth periods

**Table S1** Information on which trees were used for continuous CO_2_ flux measurements, growth measurements and non-structural carbohydrate (NSC) concentration and δ^13^C analysis.

| Tree no or locations | shoot CO_2_ influxes | stem CO_2_ effluxes | soil CO_2_ effluxes in the tree vicinity | shoot and needle growth | tracheid growth (micro-coring) | root growth in the tree vicinity | phloem NSC sampling | root NSC sampling |
| --- | --- | --- | --- | --- | --- | --- | --- | --- |
| tree 1 (cuvette tree) | May 7 ~ September 30 | May 7 ~ September 30 | May 7 ~ September 30 | April 19 ~  July 27 |  | May 7 ~ September 30 |  |  |
| tree 2 |  |  |  | April 19 ~  July 27 |  |  |  |  |
| tree 3 |  |  |  | April 19 ~  July 27 |  |  |  |  |
| tree 4 |  |  |  |  | May 7 ~ October 11 |  | May 7 ~ October 11 |  |
| tree 5 |  |  |  |  | May 7 ~ October 11 |  | May 7 ~ October 11 |  |
| tree 6 |  |  |  |  | May 7 ~ October 11 |  | May 7 ~ October 11 |  |
| tree 7 |  |  |  |  | May 7 ~ October 11 |  | May 7 ~ October 11 |  |
| tree 8 |  |  |  |  | May 7 ~ October 11 |  | May 7 ~ October 11 |  |
| random site 1 |  |  |  |  |  |  |  | May 10 ~ October 14 |
| random site 2 |  |  |  |  |  |  |  | May 10 ~ October 14 |
| random site 3 |  |  |  |  |  |  |  | May 10 ~ October 14 |


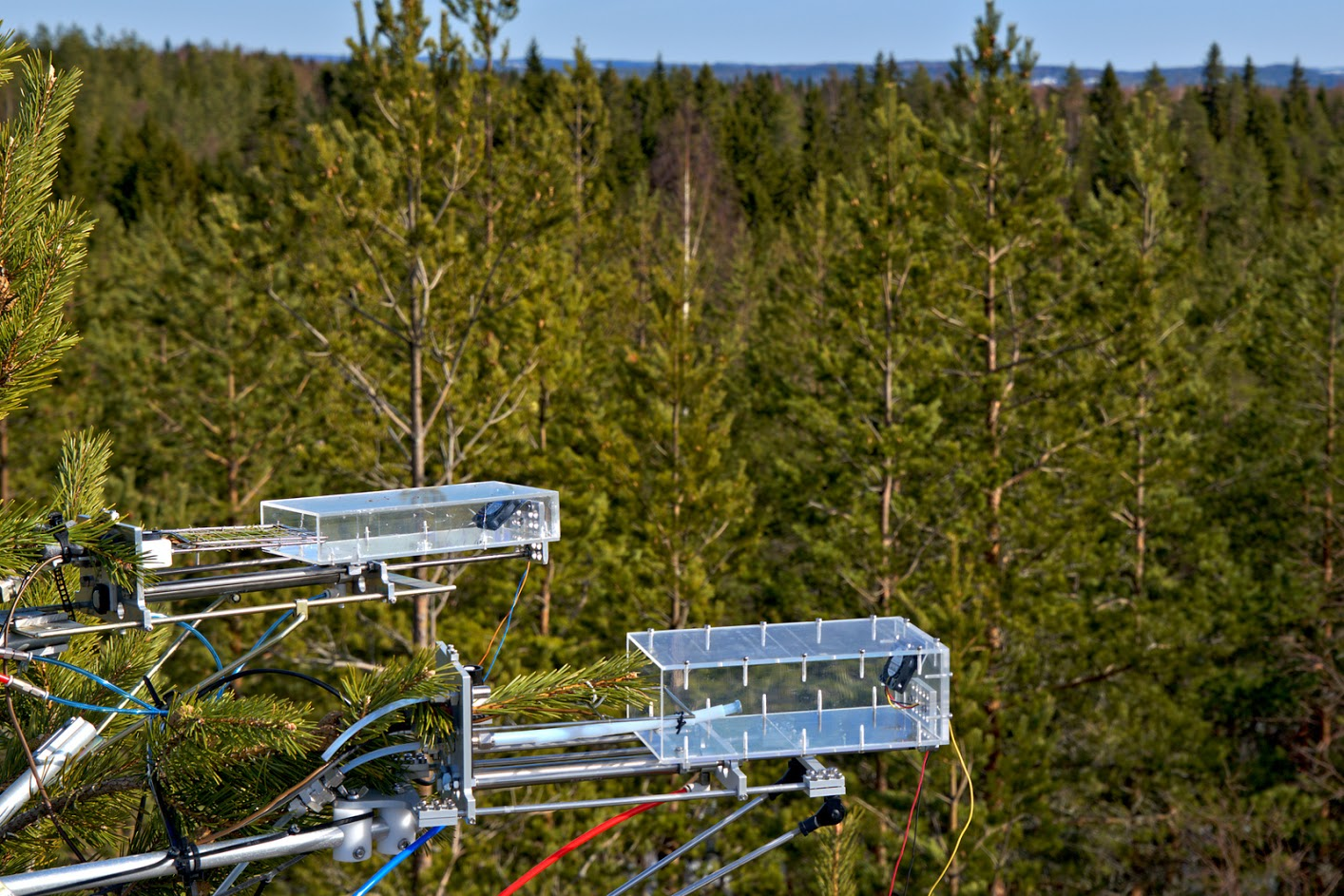


**(A)**


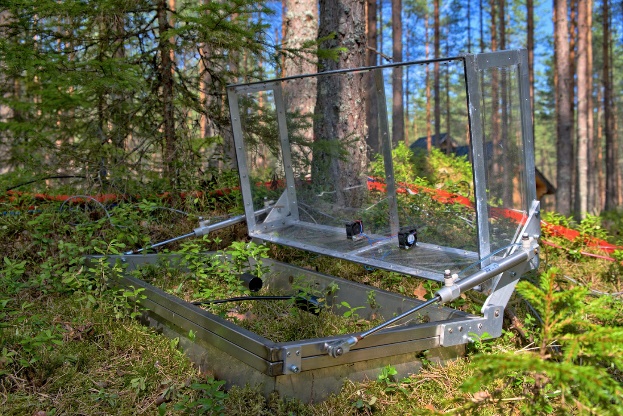


**(C)**


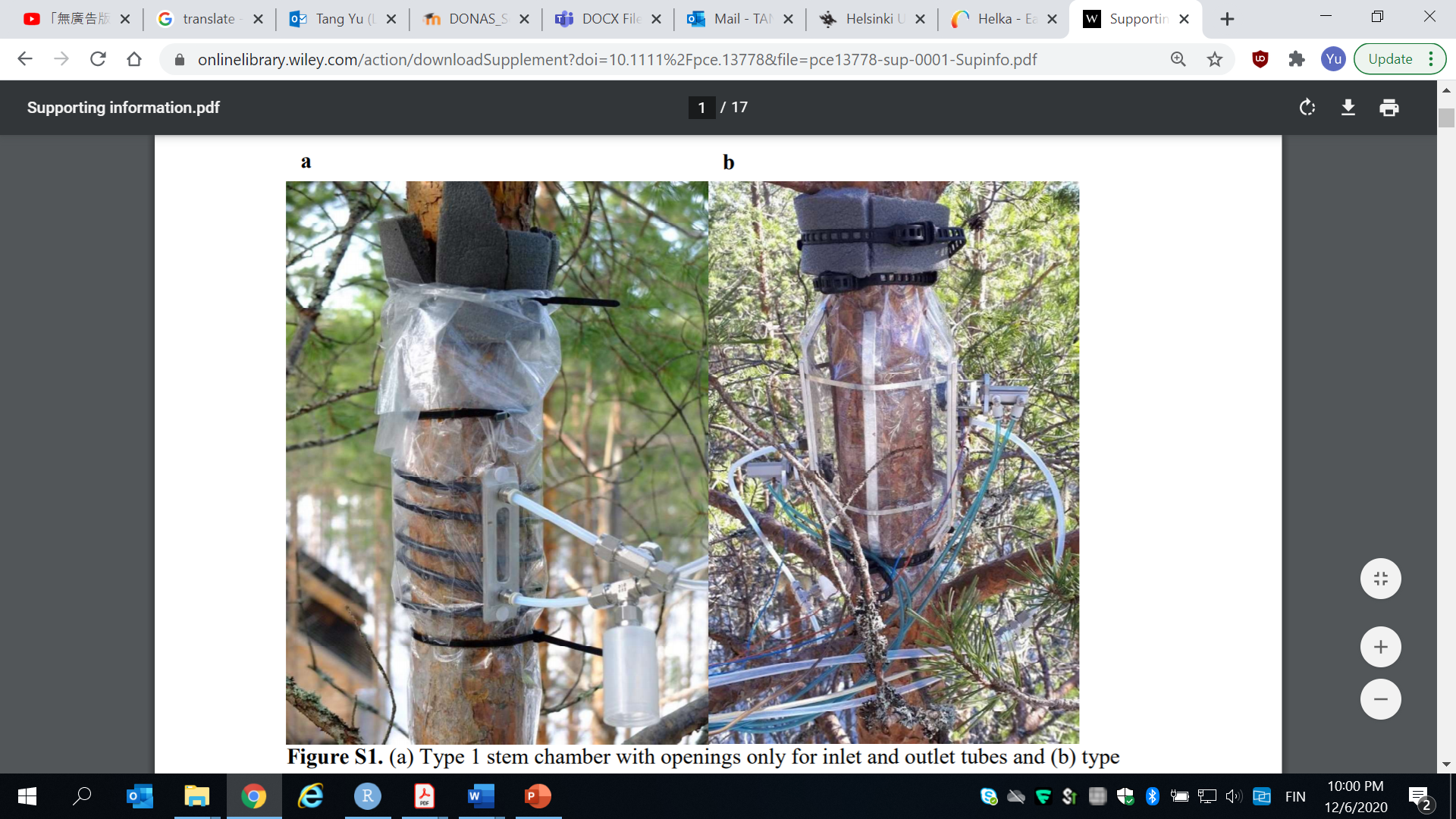


**(B)**

**Figure S1** The gas exchange measurement chambers used in this study. (A) Automated shoot chamber (photo credit to Juho Aalto); (B) Custom-made stem chamber (Rissanen et al. 2020); (C) Automated soil chamber (the same type but with vegetation inside, photo credit to Juho Aalto).

References:

Rissanen K, Vanhatalo A, Salmon Y, Bäck J, Hölttä T (2020) Stem emissions of monoterpenes, acetaldehyde and methanol from Scots pine (*Pinus sylvestris* L.) affected by tree–water relations and cambial growth. Plant Cell Environ 43:1751–1765.


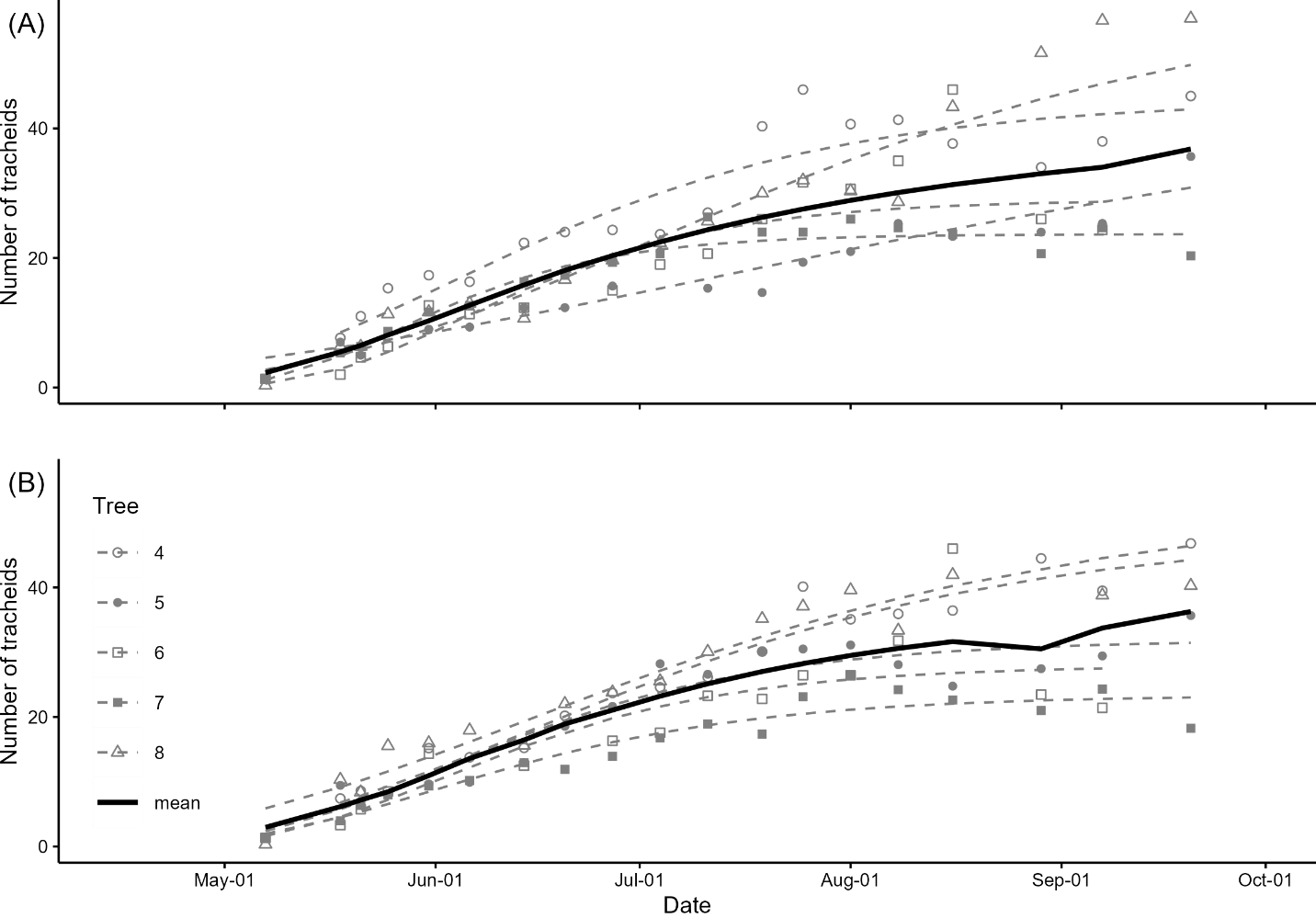


**Figure S2** Seasonal course of total number of current-year tracheids of *Pinus sylvestris* L. during the growing season of 2018 in Hyytiälä. (A) Raw data. (B) Normalized data using the tree-ring width of the previous year. Gompertz fitting curve for each tree is shown in grey dashed line. Averaged Gompertz fitting curve is shown in solid black line.


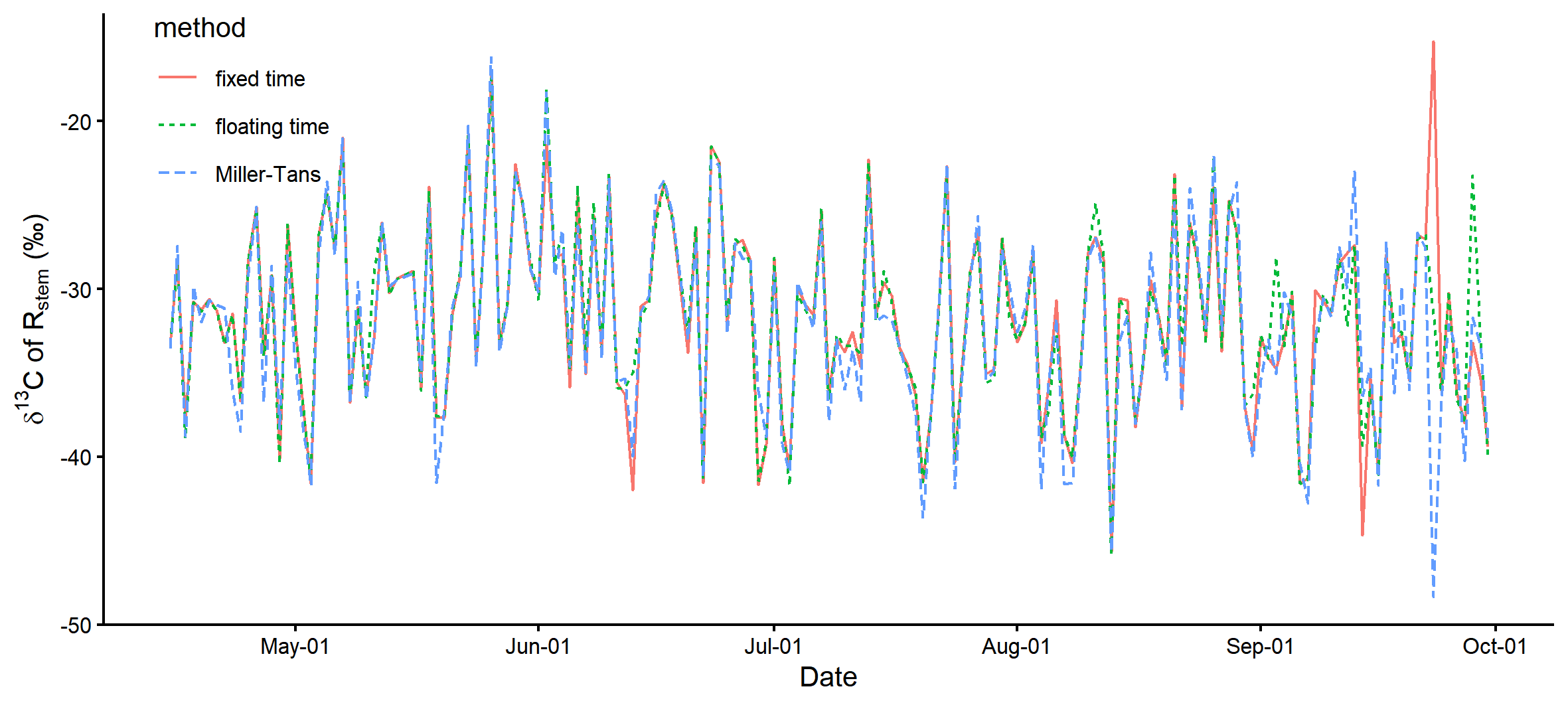


**Figure S3** Comparison of daily flux-weighed δ^13^C of stem CO_2_ efflux (R_stem_) of *Pinus sylvestris* L. during the 2018 growing season calculated from different methods. δ^13^C of R_stem_ were calculated from records taken 10–40 s after chamber closing using fixed timing (original time stamps), floating timing (time drift between the measurement system and Picarro was estimated from the measured dynamics of CO_2_, Kolari et al. 2012) and by modified Miller-Tans method (Miller and Tans 2003). Miller-Tans method was modification of the original method to address dynamic background. Initial CO_2_ concentration and δ^13^C of CO_2_ were replaced by the means of initial chamber air and replacement air weighted by their volume fractions in the chamber air as a function of time after closing. The proportion of replacement air follows exp(-flow/volume*time). The results indicate negligible difference in δ^13^C of R_stem_ across different calculation methods.

References:

Kolari P, Bäck J, Taipale R, Ruuskanen TM, Kajos MK, Rinne J, Kulmala M, Hari P (2012) Evaluation of accuracy in measurements of VOC emissions with dynamic chamber system. Atmos Environ 62:344–351.

Miller JB, Tans PP (2003) Calculating isotopic fractionation from atmospheric measurements at various scales. Tellus B: Chem Phys Meteorol 55:207–214.

**Method S1** Measurement of environmental data

Soil moisture and soil temperature in the A horizon i.e. at 2–6 cm depth in the mineral soil, were measured at 15 min interval from five locations by Campbell TDR100 Time-Domain Reflectometer and by Philips KTY81-110 temperature sensor, respectively. Precipitation above the canopy height was measured by Vaisala FD12P weather sensor at 1 min interval. Air temperature at 4.2 m height was monitored every minute with a Pt100 temperature sensor inside ventilated custom-made radiation shield.

**Method S2** Calculation of starch concentration from enzymatic hydrolysis

Aliquots of solubilized extracted starch samples as well as standards and blanks were pipetted into individual tin capsules (5×9 mm, Säntis, Teufen, Switzerland), freeze-dried and wrapped. Concentrations of starch in mg per g dry mass was calculated as follows (Eq.S1, Eq.S2):

$starch concentration=\frac{(w_{tin\_sample}-w_{tin\_blank})}{\theta\cdot w_{sample}}$ (S1)

$\theta=\frac{(w_{tin\_standard}-w_{tin\_blank})}{w_{standard}}$ (S2)

where $w_{tin\_sample}$, $w_{tin\_blank}$, and $w_{tin\_standard}$ are weight gains (in μg) in the tin capsules after pipetting the samples, blanks and standards, respectively; $w_{sample}$ and $w_{standard}$ are weights (in mg) of the samples and the standards used for starch extraction, respectively; $\theta$ is conversion factor of extracted starch mass to intact starch mass. Four blanks, two corn starch standards (Fluka, Buchs (SG), Switzerland) and two wheat starch standards (Fluka, Buchs (SG), Switzerland) were extracted with every batch of 40 samples. Average $w_{tin\_blank}$ and $\theta$ were calculated and used for each batch in the calculation. The calculation process considers (1) varying efficiencies of enzyme hydrolysis for each batch; (2) gains of blank noise and possible minor losses of starch due to sample handling during the experiment; (3) addition of water to starch molecules during hydrolysis, which leads to increased mass of extracted starch compared with intact starch.

**Method S3** Definition of tracheid production and maturation periods

To describe the progress of tracheid production or maturation in 2018, the Gompertz fitting function (Eq.S3, Zeide 1993) was applied to the number of total or mature current-year tracheid.

$y=A\cdot e^{[-e^{(\beta-kt)}]}$ (S3)

where $y$ is the number of total or mature tracheid at the observation day $t$ (day of year, DOY), $A$ is the upper asymptote defined as the number of tracheid, $\beta$ is the x-axis placement parameter of the fastest increment rate, and $k$ is the growth rate parameter determining the spread of the curve along the x-axis (Jyske et al. 2014). The Gompertz curve was fitted for each tree, as trees varied in their rates of tracheid production and maturation. Based on the Gompertz curve, the starting and ending dates for tracheid production or maturation period were defined as DOY when 5% and 90% of the number of total and mature tracheid was reached. The DOY of the maximum production and maturation rate was calculated using the parameters of the Gompertz curve (Eq.S4).

${peak}_{DOY}=\beta/k$ (S4)

Median values of DOY for the starting and ending of tracheid production or maturation were used for defining the tracheid production or maturation period of Scots pine in the study site, i.e. when half of the trees has started or completed tracheid production and maturation. The period with maximum tracheid production or maturation rate was defined as period between the second smallest ${peak}_{DOY}$ and the second largest ${peak}_{DOY}$ for tracheid production or maturation.

In addition, we decided to normalize the number of tracheids using the tree-ring width of the previous year, based on the results of Fig. **S2**. After normalization, the Gompertz fitting curves of different trees aligned better with each other.

References:

Jyske T, Mäkinen H, Kalliokoski T, Nöjd P (2014) Intra-annual tracheid production of Norway spruce and Scots pine across a latitudinal gradient in Finland. Agric For Meteorol 194:241–254.

Zeide B (1993) Analysis of growth equations. For Sci 39:594–616.

**Method S4** Definition of earlywood and latewood growth periods

To define the earlywood (EW) and latewood (LW) growth periods, the cell wall thickness and lumen diameter of the tracheid on October 11 were measured in radial direction. EW cells were defined as those whose double cell wall thickness was less than half of their radial lumen diameter (Denne 1988). For each tree, the relative position of EW to LW and the Gompertz fitting curve for tracheid maturation were used to calculate the date when EW maturation was reached. EW maturation of the observed trees was completed between July 15 and August 1. Accordingly, the EW growth period was defined as May 7 to July 14, the LW growth period as August 2 to October 11, and the EW-LW transition period as July 15 to August 1.

References:

Denne MP (1989) Definition of Latewood According to Mork (1928). IAWA J 10:59–62.
